# Imbalances in faecal and duodenal *Bifidobacterium *species composition in active and non-active coeliac disease

**DOI:** 10.1186/1471-2180-8-232

**Published:** 2008-12-22

**Authors:** Maria Carmen Collado, Ester Donat, Carmen Ribes-Koninckx, Miguel Calabuig, Yolanda Sanz

**Affiliations:** 1 Microbial Ecophysiology and Nutrition Group Institute of Agrochemistry and Food Technology (IATA), Spanish National Research Council (CSIC), PO Box 73, 46100 Burjassot, Valencia, Spain; 2 Hospital Universitario La Fe, Avenida Campanar 21, 40009 Valencia, Spain; 3 Hospital General Universitario, Avenida Tres Cruces s/n 46014 Valencia, Spain

## Abstract

**Background:**

Gut bifidobacteria are believed to influence immune-related diseases. The objective of this study was to assess the possible relationships between the gut bifidobacteria composition and coeliac disease (CD) in children.

A total of 48 faecal samples (30 and 18 samples from active and no active CD patients, respectively) and 33 duodenal biopsy specimens of CD patients (25 and 8 samples from active and non-active CD patients, respectively) were analysed. Samples (30 faecal samples and 8 biopsies) from a control age-matched group of children were also included for comparative purposes. Gut *Bifidobacterium *genus and species were analyzed by real-time PCR.

**Results:**

Active and non-active CD patients showed lower numbers of total *Bifidobacterium *and *B. longum *species in faeces and duodenal biopsies than controls, and these differences were particularly remarkable between active CD patients and controls. *B. catenulatum *prevalence was higher in biopsies of controls than in those of active and non-active CD patients, whereas *B. dentium *prevalence was higher in faeces of non-active CD patients than in controls. Correlations between levels of *Bifidobacterium *and *B. longum *species in faecal and biopsy samples were detected in both CD patients and controls.

**Conclusion:**

Reductions in total *Bifidobacterium *and *B. longum *populations were associated with both active and non-active CD when compared to controls. These bacterial groups could constitute novel targets for adjuvant dietary therapies although the confirmation of this hypothesis would require further investigations.

## Background

Coeliac disease (CD) is a chronic inflammatory disorder of the small intestine that presents in genetically predisposed individuals following gluten consumption. Gluten removal from the diet is currently the only treatment available. This disease often presents in early childhood with small intestinal villous atrophy and signs of malabsorption [1]. Recently, other factors than gluten such as imbalances in the intestinal microbiota have been reported to be associated with CD [2-5]. Most of these studies have been focused on faecal microbiota composition but less information is available on mucosa-associated microbiota of CD patients [2,5]. Neither possible relation between faecal and duodenal bacterial populations has been reported in CD.

*Bifidobacterium *genus constitutes an important bacterial group in the human gut, where this is thought to be essential to maintain health via beneficial metabolic, trophic, and protective functions [6,7]. *Bifidobacterium *is the predominant intestinal bacterial genus during the first year of life, particularly in full-term breastfed infants, although becomes quantitatively less important in adult's microbiota [8,9]. Qualitative and quantitative differences in *Bifidobacterium *species composition have been related to the development of inflammatory diseases such as allergy, irritable bowel syndrome (IBS), inflammatory bowel diseases (IBD) and colorectal cancer compared to healthy controls [10-12]. In addition, different immunomodulatory properties have been attributed to different *Bifidobacterium *species and strains that in turn were related to different disease risks. Strains of *B. adolescentis *have been shown to be more proinfammatory or had non-effect on immunity, while strains of the species *B. bifidum *and *B. longum *were shown to have immunoregulatory properties [13-15]. In this context, it has been suggested that *Bifidobacterium *strains colonizing the human gut could contribute to regulate disturbances in the balance of T-helper 1 (Th1)/Th2 lymphocyte responses to exogenous antigens related to either allergic diseases (characterized by a Th2-phenotype polarization) or Crohn and CD (characterized by a Th1 phenotype polarization). As a consequence, *Bifidobacterium *species have been regarded as particularly attractive targets for dietary intervention within the gut ecosystem to maintain intestinal homeostasis and host health.

The objective of this study was to assess the *Bifidobacterium *species composition of duodenal biopsies and faecal samples from CD patients (with active and non-active disease) and age-matched controls by the use of quantitative real-time PCR technique to elucidate their possible role in this disorder.

## Methods

### Subjects

Three groups of children were included in this study: (1) active CD patients on a normal gluten-containing diet; (2) non-active CD patients after following a gluten-free diet for at least 2 years; and (3) control children without known gluten intolerance. Biopsy specimens of the control group were obtained from children who were investigated for weight loss, growth retardation or functional intestinal disorders of non-coeliac origin, confirmed by showing a normal villous structure after diagnosis by biopsy examination.

The following faecal samples and duodenal biopsy specimens from these group of subjects were included in the analyses: 30 faecal and 25 biopsy samples from active CD patients; 18 faecal and 8 biopsy samples from non-active CD patients and 30 faecal and 8 biopsy samples from control children.

None of the children included in the study was treated with antibiotics for at least 1 month before the sampling time and they were recommended not consuming probiotic-containing products for at least 15 days prior the sampling time to limit the detection of food-related bifidobacteria without delaying too much the diagnosis procedure. The adherence to this dietary recommendation was checked at the sampling time and children that did not comply with this recommendation were not included in the study. The study protocol was approved by the local committee on ethical practice from CSIC and Hospitals taking part in the study. Children were enrolled in the study after written informed consent was obtained from their parents.

### Sampling preparation and DNA extraction

Samples were collected from every subject in sterile plastic recipients, frozen at -20°C immediately and kept at -80°C until further processing. Duodenal biopsy specimens were obtained by capsule and endoscopy after a 12-h fasting period. Faeces (1 g) and duodenal biopsy samples (10–15 mg) were weighted, diluted 1:10 (w/v) in PBS (pH 7.2) and homogenized by thorough agitation in a vortex. Aliquots of these dilutions were used for DNA extraction. DNA from both type of samples (faeces and biopsies) and from pure cultures of the different bacterial strains used as reference were extracted by using the QIAamp DNA stool Mini kit (Qiagen, Hilden, Germany) following the manufacturer's instructions.

### Real-time PCR (qPCR) analysis

Quantitative real time PCR was used to characterize the faecal microbiota by using group and species-specific primers described previously [16,17]. Briefly, PCR amplification and detection were performed with an ABI PRISM 7000-PCR sequence detection system (Applied Biosystems, UK). Each reaction mixture of 25 μl was composed of SYBR^® ^Green PCR Master Mix (SuperArray Bioscience Corporation, USA), 1 μl of each of the specific primers at a concentration of 0.25 μM, and 1 μl of template DNA. The fluorescent products were detected at the last step of each cycle. A melting curve analysis was made after amplification to distinguish the targeted PCR product from the non-targeted PCR product. Bacterial concentration from each sample was calculated by comparing the Ct values obtained from standard curves. A standard curve was made from serial dilutions of DNA isolated from each pure culture of the different reference strains. A linear relationship was observed between the cell number and CT values (r^2 ^= 0.99–0.97) when the DNA was isolated from cultures containing between 10^2 ^and 10^9 ^log cells/ml, as determined by epifluorescence microscopy counts using DAPI. The following reference strains were used to construct the corresponding standard curves: *Bifidobacterium longum *subsp. *longum *CECT 4503, *B. longum *subsp. *infantis *CECT 4553, *B. bifidum *LMG 11041, *B. breve *LMG 11042, *B. pseudocatenulatum *CECT 5776, *B. animalis *subsp. *lactis *DSMZ 10140, *B. adolescentis *LMG 11037, and *B. dentium *CECT 687. The strains were obtained from the Spanish Collection of Type Cultures (CECT), the German Collection of Microorganisms and Cell Cultures (DSMZ) and the Belgian Coordinated Collections of Microorganisms (BCCM-LMG, University of Gent).

### Statistical analyses

Statistical analyses were done using the SPSS 11.0 software (SPSS Inc, Chicago, IL, USA). Due to non-normal distribution, microbial data are expressed as medians with interquartile ranges (IQR). Comparisons among data of more than two groups of children were done by applying the Kruskal-Wallis test and comparisons between data of two groups of children were done by applying the Mann-Whitney *U *test. The possible correlation between variables was studied by applying the Spearman rank correlation coefficient and significance was established at 0.5%. The chi-square test was used to establish differences in species prevalence between the studied groups of children. A *P *< 0.050 was considered statistically significant. Bonferroni adjustment test was also applied to correct the significance for a multiple test comparisons among three groups (active and non-active CD patients and control groups), which has the advantage of reducing type I error and the disadvantage of increasing type II error.

## Results

### Subjects

Clinical characteristics of the children groups included in the study are shown in Table 1. Relative representation of males and females was almost equivalent in the study. Active CD patients were on a normal gluten-containing diet, showed clinical symptoms of the disease, positive CD serology markers (anti-gliadin antibodies AGA and anti-transglutaminase antibodies t-TG) and signs of severe enteropathy by duodenal biopsy examination classified as type 3 according to Marsh classification of CD [18]. Non-active CD patients, who had been on a gluten-free diet for at least 2 years, showed negative CD serology markers and normal mucosa or infiltrative lesion classified as type 0–1 according to Marsh classification. A total of 30 faecal samples from 56.4 months old children, and 25 biopsies from 60.6 months old children were included in the active CD patient group. A total of 18 faecal samples of 63.5 months old children and 8 biopsies of 57.8 months old children were included in the non-active CD patient group. Finally, a total of 30 faecal samples of 45.0 months old children and 8 biopsies of 49.2 months old children were included in the control group for comparative purposes.

**Table 1:** Clinical characteristics of the studied subjects.

**Characteristics**	**Active CD**	**Non-active CD**	**Control**
Number of patients	30	18	30
Age (average months and SD)	56.4 (38.5)	65.2 (37.7)	45.0 (33.5)
Gender			
- male	12/30 (40.0%)	8/18 (44.4%)	13/30 (43.3%)
- female	18/30 (60.0%)	10/18 (55.6%)	17/30 (56.7%)
Clinical			
- Abdominal	2/30 (6.6%)	-	-
- Diarrhoea	28/30 (93.4%)	2/18 (11.1%)	-
- Weight loss	9/30 (30.0%)	-	-
- Anaemia	14/30 (46.6%)	8/18 (44.4%)	-
Biochemical			
- Asymptomatic	4/30 (13.3%)	18/18 (100%)	-
- Iron deficiency	10/30 (33.3%)	-	-
Serology			
AGA (anti-gliadin antibodies)	AGA + (100%)	AGA + (0%)	AGA + (0%)
t-TG (anti-transglutaminase antibodies)	t-TG + (100%)	t-TG + (0%)	t-TG + (0%)
Duodenal Biopsy ^a^	M3 (100%)	M0-1 (100%)	M0-1 (100%)

^a ^Modified Marsh Classification of CD[18]. M0: Normal mucosa; CD highly unlikely. M01: (Infiltrative lesion): Seen in patients on a gluten-free diet (suggesting minimal amounts of gliadin are being ingested); patients with dermatitis herpetiformis (DH); and family members of patients with CD. M2 (Hyperplasic type): seen occasionally in DH. M3: > 40 Intraepithelial Lymphocytes per 100 Enterocytes, crypts increased and villi with atrophy (partial or complete villous atrophy).

### Duodenal *Bifidobacterium *species composition

*B. longum *was one of the most frequently detected species in biopsy samples followed by *B. breve*, *B. bifidum*, *B. catenulatum *and *B. lactis *(Table 2). Currently, the species *B. longum *included *B. longum *subsp. *longum*, *B. longum *subsp. *infantis *and *B. longum *subsp.* suis*, which were quantified with the set of primers for the quoted species. *B. breve *was significantly more prevalent in active CD patients than in non-active CD patients and controls although the differences were not significant (*P *> 0.05). *B. catenulatum *group was detected more frequently in controls than in active CD (*P *= 0.050) and non-active CD patients (*P *= 0.038). In addition, *B. lactis *group was detected more frequently in active CD patients (*P *= 0.003) and in controls (*P *= 0.012) than in non-active CD patients. *B. dentium *was found in active CD and non-active CD patients but not in controls. The prevalence of *B. lactis *group was also significantly different in active CD patients and controls as compared to that of non-active CD patients by applying the Bonferroni adjustment, but this was not the case for the rest of bacterial groups.

**Table 2:** Prevalence of *Bifidobacterium *group and species in faeces and duodenal biopsies of children

Microbial group in biopsy samples	Prevalence (%)^a^			*P*-value Chi-square test Bonferroni adjustment		
	
	Active CD (n = 25)	Non-active CD (n = 8)	Control (n = 8)	Active- non-active CD	Control- active CD	Control- non-active CD
*Bifidobacterium *group	100.0 (25/25)	100.0 (8/8)	100.0 (8/8)	-	-	-
*B. longum*	100.0 (25/25)	100.0 (8/8)	100.0 (8/8)	-	-	-
*B. breve*	64.0 (16/25)	37.5 (3/8)	37.5 (3/8)	0.181	0.181	0.695
*B. bifidum*	52.0 (13/25)	25.0 (2/8)	37.5 (3/8)	0.180	0.380	0.500
*B. adolescentis*	36.0 (9/25)	12.5 (1/8)	25.0 (2/8)	0.380	0.669	0.500
*B. catenulatum *group	52.0 (13/25)	37.5 (3/8)	87.5 (7/8)	0.381	0.050*	0.038*
*B. angulatum*	32.0 (8/25)	12.5 (1/8)	50.0 (4/8)	0.277	0.419	0.282
*B. lactis*	60.0 (15/25)	0.0 (0/8)	62.5 (5/8)	0.003*, ^i^	0.618	0.012*, ^i^
*B. longum *subsp. *infantis*	0.0 (0/25)	0.0 (0/8)	0.0 (0/8)	-	-	-
*B. dentium*	8.0 (2/25)	12.5 (1/8)	0.0 (0/8)	0.578	0.568	0.500

						

Microbial group in faecal samples	Prevalence (%)^a^			*P*-value Chi-square test Bonferroni adjustment		
	
	Active CD (n = 30)	Non-active CD (n = 18)	Control (n = 30)	Active- non-active CD	Control- active CD	Control- non-active CD

*Bifidobacterium *group	100.0 (30/30)	100.0 (18/18)	100.0 (30/30)	-	-	-
*B. longum*	100.0 (30/30)	100.0 (18/18)	100.0 (30/30)	-	-	-
*B. breve*	80.0 (24/30)	66.7 (12/18)	66.7 (20/30)	0.325	0.500	0.751
*B. bifidum*	100.0 (30/30)	100.0 (18/18)	100.0 (30/30)	-	-	-
*B. adolescentis*	50.0 (15/30)	83.3 (15/18)	40.0 (12/30)	0.016*, ^i^	0.452	0.045*
*B. catenulatum *group	100.0 (30/30)	100.0 (18/18)	100.0 (30/30)	-	-	-
*B. angulatum*	20.0 (6/30)	16.7 (3/18)	23.0 (7/30)	0.546	0.601	0.521
*B. lactis*	56.7 (17/30)	61.1 (11/18)	63.3 (19/30)	0.502	0.975	0.775
*B. longum *subsp. *infantis*	36.7 (11/30)	22.2 (4/18)	36.7 (11/30)	0.351	0.795	0.532
*B. dentium*	13.3 (4/30)	27.7 (5/18)	6.6 (2/30)	0.265	0.407	0.040*

^a ^Prevalence (Pr) reflects the number of positive amplifications from total samples analysed by PCR (n = number of samples analysed)

* Statistical differences were calculated by using Chi-square test 2 × 2. Significantly difference between groups was consider at *P *< 0.050

^i ^Statistical differences were corrected for a multiple comparison test (3 variable) by using Bonferroni adjustment. Significantly difference between groups was considered at *P *< 0.017.

The composition of biopsy-associated bifidobacteria assessed by qPCR is shown in Table 3. The most predominant bifidobacterial species detected in biopsy samples were *B. longum*, followed by *B. breve, B. lactis, B. bifidum *and *B. catenulatum*, whereas *B. longum *subsp. *infantis *and *B. dentium *were less prevalent.

**Table 3:** *Bifidobacterium *group and species of faeces and duodenal biopsies from children quantified by qPCR.

Microbial group in biopsy samples	Bacterial counts^a ^(Log cells/g)						*P*-value Mann-Whitney/Test Bonferroni adjustment		
	
	Active CD (n = 25)		Non-active CD (n = 8)		Control (n = 8)		Active- non-active CD	Control- active CD	Control- non-active CD
				
	Median	IQR	Median	IQR	Median	IQR			
*Bifidobacterium *group	5.95	5.55–6.21	6.15	4.97–6.28	6.27	6.03–6.80	0.604	0.009*, ^i^	0.461
*B. longum*	4.66	4.36–5.37	4.95	4.90–5.60	5.60	5.33–5.73	0.310	0.004*, ^i^	0.368
*B. breve*	5.14	4.59–5.46	3.05	3.02–3.50	5.21	5.00–5.80	0.020*	0.630	0.100
*B. bifidum*	4.35	3.40–4.75	3.98	2.15–4.44	4.17	3.48–4.66	0.800	0.700	0.950
*B. adolescentis*	3.22	2.86–3.74	3.06	-	3.87	1.80–3.30	0.600	0.327	0.667
*B. catenulatum *group	4.08	3.16–4.60	4.12	4.04–4.53	4.10	3.76–4.46	0.736	0.757	0.660
*B. angulatum*	2.95	1.54–3.80	4.10	-	3.55	1.58–4.44	0.275	0.933	0.900
*B. lactis*	6.33	5.50–6.18	-	-	5.28	4.59–5.70	-	0.033*	-
*B. longum *subsp. *infantis*	-	-	-	-	-	-	-	-	-
*B. dentium*	4.23	3.45–5.23	4.00	-	-	-	0.627	-	-

									

Microbial group in faecal samples	Bacterial counts^a ^(Log cells/g)						*P*-value Mann-Whitney test Bonferroni adjustment		
	
	Active CD (n = 30)		Non-active CD (n = 18)		Control (n = 30)		Active- non-active CD	Control- active CD	Control- non-active CD
				
	Median	IQR	Median	IQR	Median	IQR			

*Bifidobacterium *group	9.67	8.68–9.90	8.77	8.58–9.60	9.80	9.23–10.33	0.183	0.014*, ^i^	0.002*, ^i^
*B. longum*	8.90	8.56–9.40	8.30	7.78–9.00	9.28	8.88–10.10	0.030*	0.038*	< 0.001*, ^i^
*B. breve*	6.97	5.56–7.82	4.02	3.08–5.15	6.94	6.18–8.02	< 0.001*, ^i^	0.860	< 0.001*, ^i^
*B. bifidum*	7.64	6.42–8.16	6.74	6.40–6.87	6.96	6.67–7.93	0.030*	0.577	0.050*
*B. adolescentis*	6.95	5.55–7.92	5.40	4.93–7.76	5.97	5.37–6.60	0.112	0.050*	0.633
*B. catenulatum *group	7.16	6.50–8.68	7.84	7.07–8.50	7.65	7.56–8.42	0.425	0.106	0.758
*B. angulatum*	4.96	4.64–7.20	4.68	4.24–5.07	4.65	4.12–5.00	0.548	0.153	0.569
*B. lactis*	7.12	5.30–7.45	5.17	4.66–7.20	5.45	4.66–7.07	0.175	0.081	0.780
*B. longum *subsp. *infantis*	6.57	5.80–7.76	7.47	6.83–7.82	6.68	6.45–7.06	0.192	0.341	0.117
*B. dentium*	6.28	6.10–6.30	5.24	4.66–5.82	5.20	3.86–5.30	0.111	0.133	0.571

^a ^Data are shown as medians and interquartile range (IQR) of cell number per gram of faecal or duodenal biopsy sample.

* Statistical differences were calculated by using Mann-Whitney *U *test comparing two variables. Significantly difference between groups was considered at *P *< 0.050.

^i ^Statistical differences were corrected for a multiple comparison test (3 variable) by using Bonferroni adjustment. Significantly difference between groups was considered at *P *< 0.017.

Significant differences were detected by using the Kruskal-Wallis test among active and non-active CD patient and control groups for total *Bifidobacterium *(*P *= 0.040), *B. longum *(*P *= 0.017), *B. breve *(*P *= 0.018) and *B. bifidum *(*P *= 0.022). No differences were found for the other analysed species. Comparisons of bifidobacterial levels between groups by using the Mann-Whitney *U *test allowed the detection of significantly higher levels of total *Bifidobacterium *in controls than in active CD patients (*P *= 0.009), although no significant differences were found between non-active CD patients and controls (*P *= 0.461). *B. longum *levels were also significantly higher in controls than in active CD patients (*P *= 0.004) and slightly higher than in non-active CD patients although not significantly (*P *= 0.368). Total *Bifidobacterium *and *B. longum *group levels were also significantly different between active CD and control children by applying the Bonferroni adjustment. *B. breve *levels were significantly lower in non-active CD patients than in active CD patients (*P *= 0.020) and also slightly lower (*P *= 0.100) than in control children as it was the case for faeces. A similar trend was found for *B. bifidum *but none of the differences reached statistical significance. *B. lactis *levels were higher in active CD patients than in controls (*P *= 0.033), while this species was not detected in non-active CD patients.

### Faecal *Bifidobacterium *species composition

The number of positive samples for *Bifidobacterium *group and species detected by PCR (prevalence) compared to the total number of samples tested in the study are shown in Table 2. *B. longum*, *B. bifidum *and *B. catenulatum *groups were detected in all faecal samples, whereas the other *Bifidobacterium *species analysed were not detected in every sample (Table 2). *B. breve *was detected more frequently in active CD patients than non-active CD patients and controls, although the differences were not significant (*P *> 0.05). *B. adolescentis *was detected more frequently in non-active CD patients than in active CD patients (*P *= 0.016) and controls (*P *= 0.045). *B. dentium *was significantly more prevalent in non-active CD patients than in controls (*P *= 0.040), and the same trend was detected between active CD patients and controls but the differences were not statistically significant. *B. adolescentis *prevalence was also significantly different between non-active CD patients and active CD patients (*P *= 0.016) by applying the Bonferroni adjustment test. No significant differences were found for the other *Bifidobacterium *groups or species.

The bacterial composition of faecal samples from the three groups of children under study assessed by qPCR is shown in Table 3. The most predominant *Bifidobacterium *groups present in faecal samples were *B. longum, B. catenulatum *group and *B. bifidum*. Significant differences were obtained by using the Kruskal-Wallis test among active and non-active CD patient and control groups for total *Bifidobacterium *(*P *= 0.002), *B. longum *(*P *< 0.001), *B. breve *(*P *< 0.001), *B. bifidum *(*P *= 0.030) and *B. adolescentis *(*P *= 0.020). No differences were found for the other studied species. The comparison of bifidobacterial levels between groups by using the Mann-Whitney *U *test allowed the detection of significant differences in several cases. Total *Bifidobacterium *levels were significantly higher in control samples than in those of active CD (*P *= 0.014) and non-active CD patients (*P *= 0.002). No differences were found between active and non-active CD patients (*P *= 0.183). *B. longum *levels were significantly higher in controls than in active CD (*P *= 0.038) and non-active CD patients (*P *< 0.001); moreover, *B. longum *levels were significantly higher in non-active CD patients than in active CD patients (*P *= 0.030). Most of these differences were also statistically significant by applying the Bonferroni adjustment at *P *< 0.017 (Table 3). *B. breve *levels were significantly higher in active CD (*P *= 0.001) and control children (*P *< 0.001) than in non-active CD patients, which showed the lowest counts of this species, but differences between active CD patients and controls (*P *= 0.860) were not found by either Mann Whitney or Bonferroni adjustment test. Similarly, *B. bifidum *levels were higher in active CD patients (*P *= 0.030) and controls (*P *= 0.050) than in non-active CD patients, whereas significant differences were not found between active CD and control children (*P *= 0.577). *B. adolescentis *levels were significantly higher (*P *= 0.050) in active CD patients than in controls while differences were not found neither between active and non-active CD patients nor between non-active CD patients and controls. However, differences in *B. bifidum *and *B. adolescentis *were not statistically significant when applying the Bonferroni adjustment test. No differences were found for any other *Bifidobacterium *species analysed between the three groups of children under study (Table 3).

### Relationships between duodenal and faecal *Bifidobacterium *species composition

**Figure 1:**
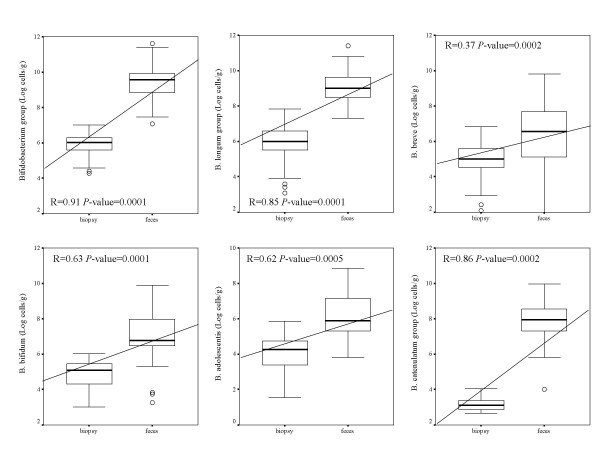
**Correlations of bifidobacterial groups of faecal and duodenal biopsy samples from all children (active and non-active CD patients and controls) under study**. Data represent the positive samples. The line in the box is the median (50% percentile), with the lower line the lower 25% border (25% percentile) and the upper line the 75% (75% percentile) border. The end of the upper vertical line is the maximum data value, outliers not considered. The end of the lower vertical line is the lowest value, outliers not considered. The separate dots or asterisks indicate outliers. Significant differences (*P *< 0.050) were found between faeces and biopsy levels of every bifidobacterial group analysed when considering all subjects.

Faecal samples showed higher numbers (*P *< 0.050) of bifidobacteria than duodenal biopsies samples for every analyzed group, as anticipated (Fig. 1). Correlations were generally found between levels of each bifidobacterial group detected in faecal and biopsy samples within the same individual. Thus, low faecal bifidobacterial levels corresponded to low biopsy bifidobacterial levels in the same subjects and vice versa. Correlations between total *Bifidobacterium *levels in faecal and biopsy samples were significant in active CD patients (R = 0.84, *P *< 0.001), non-active CD patients (R = 0.67, *P *= 0.001) and controls (R = 0.68, *P *< 0.001). Similarly, correlations between *B. longum *levels in faecal and biopsy samples were significant in active CD patients (R = 0.80, *P *< 0.001), non-active CD patients (R = 0.79, *P *< 0.001) and controls (R = 0.53, *P *= 0.001). In active CD patients, correlations were also found for *B. breve *(R = 0.44, *P *= 0.001), *B. bifidum *(R = 0.52, *P *= 0.001), *B. adolescentis *(R = 0.57, *P *= 0.002), *B. catenulatum *(R = 0.70, *P *< 0.001) and *B. lactis *(R = 0.70, *P *< 0.001), whereas no correlations were found for *B. angulatum, B. longum *subsp.*infantis *and *B. dentium*. In controls, significant correlations between bifidobacterial levels in faeces and biopsy samples were also found for *B. catenulatum *group (R = 0.40, *P *= 0.017) and *B. longum *subsp.*infantis *(R = 0.54, *P *= 0.030) and in non-active CD patients for *B. bifidum *(R = 0.54, *P *= 0.012) and *B. catenulatum *group (R = 0.70, *P *= 0.001).

## Discussion

*Bifidobacterium *species are regarded as key biological markers of a healthy gut. Herein, features of the composition of this bacterial genus in the gut ecosystem of CD patients, with active and non-active disease, and control children have been reported for the first time, and the existing correlations between faecal and duodenal biopsy-associated bifidobacteria. Active and non-active CD patients showed lower numbers of total *Bifidobacterium *in both types of samples analysed, faeces and duodenal biopsy specimens. These differences were significant in every case except for biopsy samples of non-active CD patients and controls. A similar trend was obtained by using the Bonferroni adjustment test although differences in faecal *B. longum *levels between control children and active CD patients were not significant. Faecal imbalances in total *Bifidobacterium *levels were detected in both active and non-active CD patients as compared to controls and therefore they seemed to be irrespectively of disease activity. However, duodenal *Bifidobacterium *levels seemed to be partially restored after the gluten-free diet since significant differences were not found between non-active CD patients and control children. These different trends could be a consequence of the limited number of biopsy samples available when compared with faecal samples. *Bifidobacterium *numbers of the mucosa of IBD patients and allergic infants were also found to be reduced compared to controls [10,19,20]. Some reports also showed that allergic infants were colonized by bifidobacteria less often and at lower concentrations than controls [9,10,21,22]. A significant reduction of gut bifidobacterial levels was also reported to precede the development of atopic diseases, indicating a relation between relative abundance of this bacterial genus and the development of immune-related disorders [10]. A recent report also indicated that high numbers of bifidobacteria may correlate positively with the normalization of inflammatory status and improved glucose tolerance and glucose-induced insulin secretion in an obesity animal model induced by a high-fat containing diet [23]. These findings, together with the present results, suggest that lower numbers of total bifidobacteria may be associated with inflammatory processes, supporting the hypothesis that bifidobacteria are required to maintain intestinal homeostasis.

The most predominant bifidobacterial groups detected in both, biopsies and faeces, were *B. longum, B. bifidum *and *B. catenulatum *followed by *B. breve *and *B. lactis*. In agreement with previous studies, *B. longum *was the species most commonly found in the faecal and duodenal mucosa-associated microbiota [9,12]. The levels of *B. longum *were markedly lower in active CD patients and to a lesser extent in non-active CD patients than in controls in both faecal and biopsy samples according with the data obtained for total *Bifidobacterium*; the differences were significant in every case except for biopsy samples of non-active CD patients and controls presumably due to their limited number. Imbalances in *B. longum *levels were found irrespectively of the phase of the disease (active or non-active) particularly in faeces; however, the gluten-free diet could also influence the levels of this species since differences were found between active and non-active CD patients. Duodenal *B. longum *levels seemed to be partially restored after the gluten-free diet, following the same trend as that detected for total *Bifidobacterium *levels.

Bifidobacteria have been demonstrated to have a species and strain-specific influence on immunity [14,15,24]. Strains of the genus *Bifidobacterium *have been shown to polarized Th2/Th1 responses in a specific manner, thereby modulating unbalanced cytokine production characteristic of either Th2-type (e.g. allergy) or Th1-type diseases (e.g. Crohn disease and CD) and overall inflammation [13,25]. It has been speculated that typical adult-type bifidobacterial species such as *B. adolescentis *and *B. catenulatum *group could favour Th2-biased immune responses characteristic of allergy inflammation [14]. In contrast, anti-inflammatory properties have been generally attributed to strains of the species *B. longum *mainly related to their ability to stimulate regulatory cytokine production (e.g. IL-10) [25,26]. In this study, the higher levels of *B. longum *detected in control samples (biopsies and faeces) compared to those found in active and non-active CD patient samples suggest that this bifidobacterial group could exert a protective role in CD. Otherwise, changes in the intestinal environment of CD patients, such as the mucus layer composition, could secondarily lead to changes in gut bacterial populations. In this context, lower levels of *B. longum *have also been reported in IBD and colorectal cancer patients [12].

*B. breve *and *B. bifidum *numbers were particularly reduced in non-active CD patients when compared with active CD-patients and controls in both biopsy specimens, and especially in faecal samples, indicating that these reductions could be due to the gluten-free diet rather than to the disease.

*B. adolescentis *were detected in slightly higher numbers in faecal samples of active CD patients than in controls and its prevalence was also higher in faeces of non-active CD patients than in those of controls. However, this loosely association between *B. adolescentis *and CD was neither confirmed in biopsies nor in a preliminary study carried out previously in a few faecal samples of active CD patients and controls by PCR-DGGE [7].

In general, this study confirms that the dominant bifidobacterial species detected in faeces represented those found in duodenal biopsies although in different quantities (Fig. 1), supporting previous reports on adults and infants [27,28]. Significant correlations were detected between levels of total *Bifidobacterium *and the species *B. longum *in faecal and biopsy samples, which were the bacterial groups most clearly related to CD. Therefore, faecal alterations of *Bifidobacterium *and *B. longum *levels reflect those occurring in the duodenum. These could be used as indexes of CD in faeces without the use of invasive biopsy techniques, although further studies should be carried out in other population groups to confirm such hypothesis.

## Conclusion

Active and non-active CD is associated with changes in number, composition and prevalence of *Bifidobacterium *populations. The microbiota of CD patients is characterised by reductions in total *Bifidobacterium *and *B. longum *numbers. These microbial deviations are not completely restored after treatment with a gluten-free diet. Thus, the results suggest that total and specific *Bifidobacterium *species could be possible protective factors for CD. Therefore, the administration of specific probiotics and prebiotics to increase their intestinal levels could constitute a possible adjuvant therapeutic strategy for this disorder. Confirmation of such hypothesis would require further investigations.

## Authors' contributions

The author's responsibilities were as follows: YS. made the microbiological study concept and design, MCC acquired microbiology data and made the statistical analyses. MC, CR-K and ED acquired clinical data. All authors participated in preparation of the manuscript and approved the final version. None of the authors has conflict of interests.
